# Carbon nanotube/metal-sulfide composite flexible electrodes for high-performance quantum dot-sensitized solar cells and supercapacitors

**DOI:** 10.1038/srep46519

**Published:** 2017-04-19

**Authors:** Chandu V. V. Muralee Gopi, Seenu Ravi, S. Srinivasa Rao, Araveeti Eswar Reddy, Hee-Je Kim

**Affiliations:** 1 School of Electrical Engineering, Pusan National University, Gumjeong-Ku, Jangjeong-Dong, Busan 46241, South Korea; 2 Department of Chemical Engineering, Inha University, Incheon, 22212, South Korea

## Abstract

Carbon nanotubes (CNT) and metal sulfides have attracted considerable attention owing to their outstanding properties and multiple application areas, such as electrochemical energy conversion and energy storage. Here we describes a cost-effective and facile solution approach to the preparation of metal sulfides (PbS, CuS, CoS, and NiS) grown directly on CNTs, such as CNT/PbS, CNT/CuS, CNT/CoS, and CNT/NiS flexible electrodes for quantum dot-sensitized solar cells (QDSSCs) and supercapacitors (SCs). X-ray photoelectron spectroscopy, X-ray diffraction, and transmission electron microscopy confirmed that the CNT network was covered with high-purity metal sulfide compounds. QDSSCs equipped with the CNT/NiS counter electrode (CE) showed an impressive energy conversion efficiency (η) of 6.41% and remarkable stability. Interestingly, the assembled symmetric CNT/NiS-based polysulfide SC device exhibited a maximal energy density of 35.39 W h kg^−1^ and superior cycling durability with 98.39% retention after 1,000 cycles compared to the other CNT/metal-sulfides. The elevated performance of the composites was attributed mainly to the good conductivity, high surface area with mesoporous structures and stability of the CNTs and the high electrocatalytic activity of the metal sulfides. Overall, the designed composite CNT/metal-sulfide electrodes offer an important guideline for the development of next level energy conversion and energy storage devices.

The energy crisis and global warming concerns have increasing the demand for progress into high performance energy conversion and energy storage devices. The development of new electrode materials for energy conversion and energy storage technologies, particularly quantum-dot-sensitized solar cells (QDSSCs) and supercapacitors have attracted special attention. As third-generation energy conversion devices, QDSSCs are considered promising substitutes to dye-sensitized solar cells (DSSCs), owing to the excellent properties of QDs instead of dye molecules, such as band gap tunability, high absorption coefficient, hot carrier extraction, multiple exciton generation, solution processability, and low-cost facile preparation^1^,^2^,^3^,^4^,^5^,^6^. There are many circumstances that significantly affect the QDSSCs performance, such as the QD sensitizers, the electrolyte, the cell structure, and the counter electrode (CE). Among them, the CE has been less focused than the other factors, even though this may be the key to developing efficient QDSSCs. Besides, extensive efforts have been made to develop quantum dot sensitizers^7^,^8^ and electrolyte, QDDSCs show poorer performance than DSSCs^9^,^10^. One of the major hurdles is the lower conductivity (i.e., sheet conductivity) and poor electrocatalytic activity toward electrolyte regeneration, which increase the charge transfer resistance at the CE/electrolyte interface and decrease the fill factor and energy conversion efficiency of QDSSCs^11^. However, it is important to optimize and design a new CE catalyst with stable, high catalytic activity, and superior conductivity to elevate the performance of QDSSCs.

In traditional DSSCs, a platinum (Pt) CE is a widely used CE material. On the other hand, its use in conjunction with a polysulfide electrolyte is inappropriate in QDSSCs owing to the suppression of conductivity and surface activity of Pt electrodes caused by the chemisorption of sulfur ions in the electrolyte^12^,^13^. Therefore, other CE materials, such as PbS, Cu_2_S, CoS, CoS/CuS, NiS, carbon, ZnO/metal sulfides, and Cu_2_S-reduced graphene oxide composites, have been inspected for their higher electrocatalytic activity towards the reduction of the polysulfide redox couple^14^,^15^,^16^,^17^,^18^,^19^,^20^,^21^. Carbon nanotubes (CNTs) are attractive candidates with high electrical conductivity, large surface area, and low density, making them candidates for applications in various fields, such as microelectronics, energy, and biotechnology^22^,^23^,^24^,^25^,^26^. Recently, multiwall carbon nanotubes (MWCNTs) were proposed as an efficient CE material in QDSSCs to suppress the charge transfer resistance at the CE/electrolyte interface and achieve higher efficiency (4.67%) than CuS CE (3.67%)^27^. Luo *et al*. developed a tunable size and thick CNT film by the layer-by-layer condensation of a cylindrical CNT assembly^28^. Zeng *et al*. produced MWCNT-CZTSe composite films using a spray deposition method and used them as highly effective CEs in a QDSSC system with an optimized efficiency of 4.60%^29^. Seoul *et al*. developed a composite electrode by integrating carbon nanotube (CNT)-reduced graphene oxide (RGO) with Mo-compound particles (Mo_2_N, Mo_2_C, and MoS_2_) supports for use as an efficient catalyst in QDSSCs and obtained a high efficiency of 5.41%^30^.

Carbon materials^31^,^32^,^33^,^34^,^35^, metal oxides/hydroxides^36^,^37^, and conducting polymers^38^,^39^ also have been considered as electrodes in SCs. Therefore, many research efforts have been made to improve the specific capacitance (C_s_) of SC electrode materials^40^. Recently, transition-metal chalcogenides also have gained tremendous interest as materials for SCs owing to their high performance, cost-effectiveness, and abundance^41^. Shen *et al*. proposed a rational design and fabrication of NiCo_2_S_4_ nanosheets supported on nitrogen-doped carbon foams (NCF) because it delivers an ultrahigh capacitance of 877 F g^−1^ and excellent cycling stability^42^. Zhu *et al*. fabricated composite CNTs with CuS nanoneedles that exhibited a capacitance of 122 F g^−1^ in a KOH electrolyte^43^. Recently, Tong *et al*. developed an asymmetric supercapacitor using positive and negative electrodes of Zn_0.76_Co_0.24_S/NGN/CNTs film NGN/CNTs film, respectively, resulting in a high energy density and superior cycling stability^44^. To date, there have been many reports on individual CNTs and metal chalcogenides used as electrode materials in both QDSSCs and SCs. None of these studies, however, examined composite CNT/metal sulfides as efficient electrode for high-performance QDSSCs and SCs. Therefore, the combination of CNTs with metal sulfides has been proposed to enhance the electrocatalytic activity and conductivity of CEs.

In the present study, a set of advanced composite electrode materials were developed by combining metal sulfides (PbS, CuS, CoS, and NiS) with multi-walled carbon nanotubes (CNTs) supports for use as efficient catalysts in QDSSCs and SCs. The combination of CNT/metal-sulfides delivers an impressive electron pathway because of the large electrical conductivity of the CNT network and that metal sulfides enhance the electrochemical performance owing to their electrocatalytic behavior. The morphology and chemical structure of the synthesized catalysts were examined by high resolution electron microscopy and physiochemical analysis. For the capacitance measurements, two electrode symmetric supercapacitors were prepared and their capacitances were measured using galvanostatic charge–discharge techniques.

## Results and Discussion

**Figure 1:**
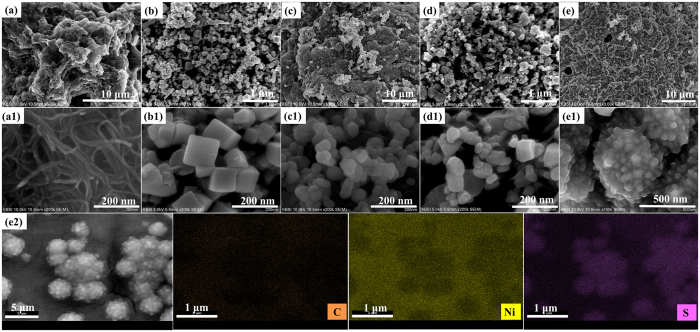
Low magnification (**a–e**) and high-magnification (a1-e1) HRSEM images of CNT (**a**,a1), CNT/PbS (**b**,b1), CNT/CuS (**c**,c1), CNT/CoS (**d**,d1) and CNT/NiS (**e**,e1) films on Ni-foam substrate. (e2) EDX-SEM image and corresponding elemental mapping of C, Ni, and S elements in CNT/NiS electrode.

**Figure 2:**
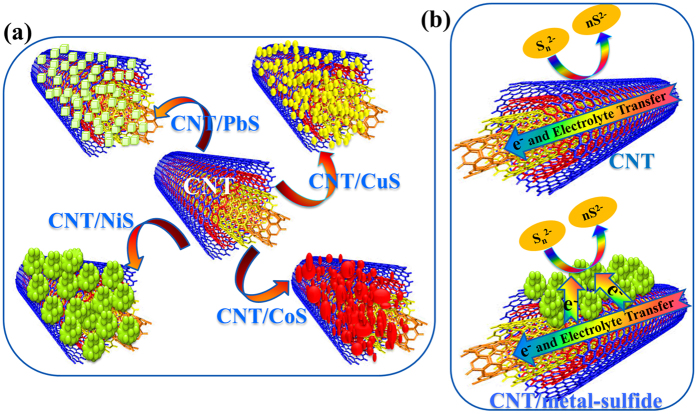
(**a**) Schematic representation of CNT/metal-sulfides. (**b**) Comparison of electron transfer process in bare CNT and CNT/metal-sulfide electrode material in presence of polysulfide electrolyte.

The morphology of the carbonaceous materials with metal sulfides (CNT/PbS, CNT/CuS, CNT/CoS, and CNT/NiS) on Ni-foam were confirmed by FESEM. As shown in Figure 1(a,a1), the CNTs were randomly entangled with an outer diameter of approximately 10 nm. Figure 1(b,b1) presents the surface morphology of the CNT/PbS composite. During the deposition of PbS on CNT, the PbS nanocubes were deposited uniformly on the surface of the CNTs with a diameter of 125 nm. Figure 1(c,c1) and 1(d,d1) shows the nanoparticle morphology of CuS and CoS deposited on the CNT surface. The diameters of the CuS and CoS nanoparticles on CNT’s were in the range of 38–63 nm and 36–94 nm, respectively. On the other hand, uniform agglomerated NiS nanoparticles were observed on the CNT surface (Fig. 1(e,e1)). A uniform dispersion of NiS aggregates on the CNTs increased the number of reactive sites for the reaction. As a result, the void spaces between the CNTs were filled with metal sulfides that formed a network wrapping the CNTs. This resulted in improved electronic conductivity between the metal sulfides and CNT network, yielding more defects and an enhancement of the charge transport process compared to the bare CNT material. Furthermore, EDX and elemental mapping (Fig. 1(e2)) was used to identify the uniformity of the elemental distribution in CNT/NiS sample, where Ni and S are decorated uniformly and densely on the surface of the CNTs, denoting that the Ni and S atoms are well deposited on the CNT structure, the resulting C content is lower than that of the other elements. Figure 2(a) presents a schematic representation of the CNT/metal-sulfides. The electrons in CNT/metal-sulfides find the shortest path to accelerate charge transport and facilitate reduction of the polysulfide electrolyte compared to the bare CNT network, which encourages the enhanced electrocatalytic activity of the composite electrode material to enable higher QDSSC and SC performance (Fig. 2(b)).

**Figure 3:**
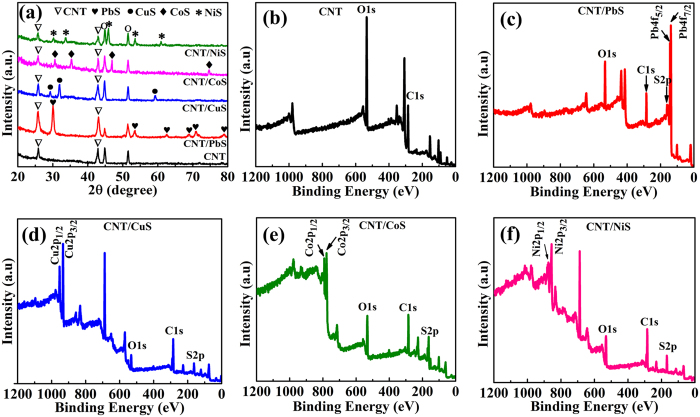
(**a**) XRD patterns of CNT and CNT/metal-sulfide composites on Ni-foam. XPS survey spectra of (**b**) CNT, (**c**) CNT/PbS, (**d**) CNT/CuS, (**e**) CNT/CoS, and (**f**) CNT/NiS films on Ni-foam.

Figure 3(a) depicts the XRD patterns of the CNT and CNT/metal-sulfide composites. The XRD patterns of the CNT and CNT/metal-sulfide composite show typical peaks at 24° and 44° 2θ, corresponding to the (002) and (100) crystal planes, respectively, confirming that the samples have a graphitic structure (Joint Committee for Powder Diffraction Studies (JCPDS) No. 01–0646). In addition to the CNT peaks, the CNT/PbS shows the additional peaks of PbS at 30.1°, 53.4°, 62.5°, 68.8°, 70.9° and 78.8° 2θ, corresponding to the (200), (222), (400), (331), (420), and (422) lattice planes of the cubic PbS phase (JCPDS No. 05-0592). The CNT/CuS electrode shows the peaks for CuS at 29.3°, 31.8°, and 59.3° 2θ, which were assigned to the crystal planes (102), (103), and (116) of the hexagonal CuS phase (JCPDS No. 01-079-2321). The hexagonal phase CoS peaks are observed on the CNT surface at 30.6° (100), 35.3° (101), 46.9° (102), and 74.6° (202), which are well matched to the JCPDS No. 03-065-3418. The XRD pattern of the CNT/NiS exhibiting XRD peaks at 30.2°, 33.7°, 45.9°, 53.5°, and 61.0° are for the hexagonal NiS planes of (100), (002), (102), (110), and (103), respectively (JCPDS No. 01-075-0613). The composite samples have both the peaks of CNTs and metal sulfides, indicating their good crystallinity and improved electronic conductivity compared to the bare CNT sample.

The CNT/metal-sulfide composites were characterized further by XPS. Figure 3(b–f) depicts the XPS survey spectra of CNT, CNT/PbS, CNT/CuS, CNT/CoS, and CNT/NiS, respectively. The survey spectra of all the electrodes affirm the existence of C and the presence of trace amounts of O in all films is correlated with moisture or oxygen molecules. The high resolution XPS spectra of C, Pb, Cu, Co, Ni and S elements in all electrodes are shown in Fig. S1†. Figure 3(c) shows the two main peaks of Pb 4 f located at 137.4 and 142.3 eV, which were assigned to Pb 4f_7/2_ and Pb 4f_5/2_, respectively. In the case of the XP spectrum of CNT/CuS (Fig. 3(d)), the Cu 2p_3/2_, and Cu 2p_1/2_ peaks were detected at 932.4 and 952.7 eV, respectively, and the CNT/CoS spectrum in Fig. 3(e) clearly reveals the intense Co 2p_3/2_ and Co 2p_1/2_ peaks at 779.5 eV and 794.6 eV, respectively. As shown in Fig. 3(f), the two main peaks separated by 18.2 eV at 856.1 eV and 874.3 eV correspond to Ni 2p_3/2_ and Ni 2p_1/2_, respectively. All samples in Fig. 3(b–f) exhibiting a peak of S 2p at 162.3 eV were indexed to sulfide. Table S1 (Supporting Information) lists the amounts of elements that exist in all the CNT/metal-sulfide samples. The C content decreased with the further deposition of metal sulfides on the CNT surface. Interestingly, a lower C content was observed in the CNT/NiS sample, suggesting that the residual NiS was well enclosed inside and on the surface of the CNT matrix to enable higher electrocatalytic activity. In addition, the higher sulfur content in CNT/NiS will lead to more defects in the form of metal vacancies, resulting in an increase in the electrical conductivity and catalytic activity of CE. The XPS results were unambiguous and corroborated that the metal sulfides had been coated successfully on the CNT surface.

**Figure 4:**
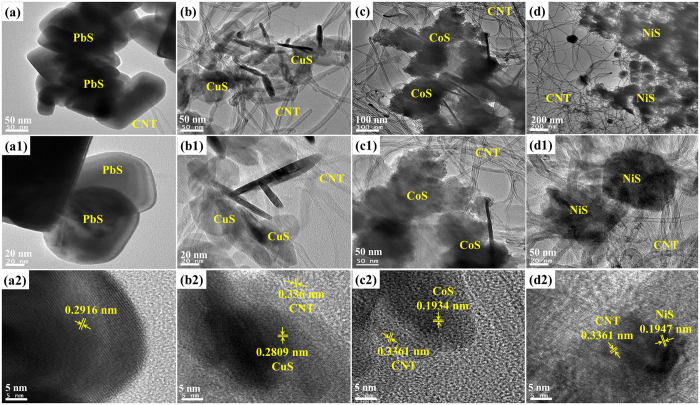
TEM images of CNT/PbS (**a**,a1,a2), CNT/CuS (**b**,b1,b2), CNT/CoS (**c**,c1,c2) and CNT/NiS composites (**d**,d1,d2) and their respective enlarged TEM (a1-d1) and HRTEM (a2-d2) images.

Detailed structural and morphological studies of the CNT/metal-sulfides were examined by FE-TEM and the corresponding low-magnified and high-magnified TEM images are shown in Fig. 4. In addition, the crystal structures of the CNT/metal-sulfides were crosschecked by measuring the lattice spacing. TEM showed well-dispersed CNTs with a diameter of 20 nm in all CNT/metal-sulfides. Figure 4(a,a1) shows that the PbS nanocubes were spread over the CNT surface with lattice fringes with a d spacing of 0.2916 nm (Fig. 4(a2)), which was indexed to the (200) plane of PbS. The CuS/CNT composite (Fig. 4(b,b1)) showed a conductive CNT network coated with CuS nanoparticles. Figure 4(b2) shows that the lattice spacing’s are 0.2809 nm and 0.336 nm, corresponding to the (103) and (002) crystal planes of the CuS and CNT phase, respectively. The nanoparticle-like CoS structures were assembled on surface of the CNTs in the CNT/CuS composite (Fig. 4(c,c1)) and the d-spacing extracted from the lattice fringes were 0.1934 nm and 0.33361 nm (Fig. 4(c2)), which were indexed to the (102) and (002) planes of the CoS and CNT phases, respectively. Figure 4(d,d1) shows the agglomerated NiS nanoparticles with CNTs and the size corresponds approximately to that indicated by the SEM images. The high resolution TEM image of CNT/NiS in Fig. 4(d2) shows an interplanar spacing of 0.3361 nm and 0.1947 nm corresponding to the (002) and (102) planes of the CNT and NiS phase, respectively. Therefore the TEM images of CNT/metal-sulfides are consistent with the SEM and XRD analyses.

**Figure 5:**
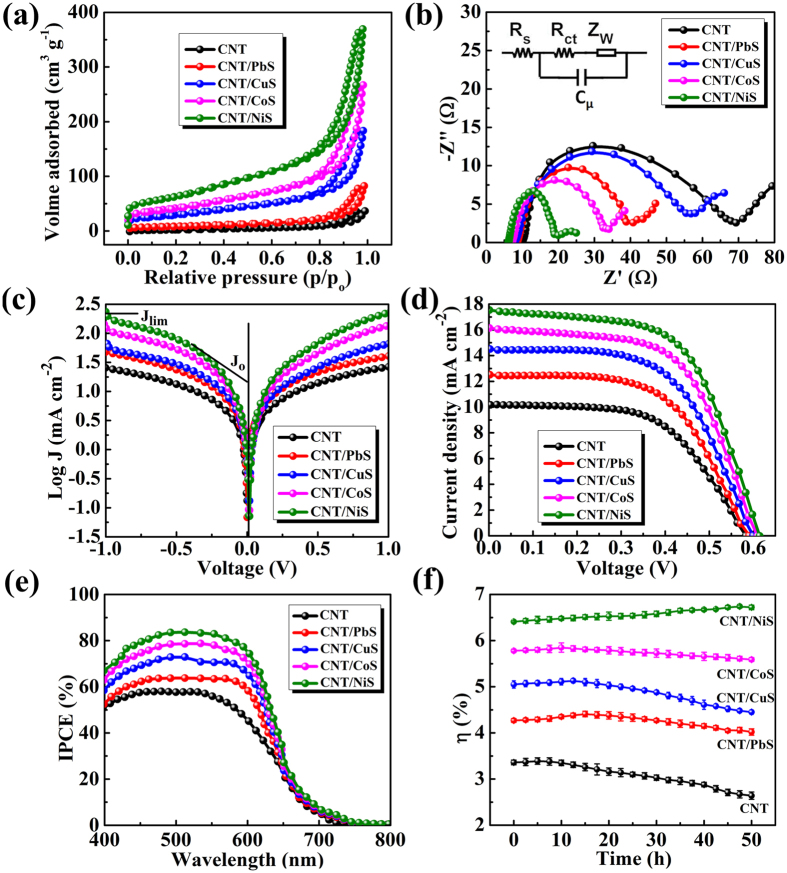
(**a**) BET surface area characteristics for the CNT/metal-sulfide electrodes. (**b**) Nyquist plots and (**c**) Tafel polarization curves of symmetric cells using the corresponding CNT/metal-sulfide CEs. Inset in (**c**) is equivalent circuit for fitting EIS plots. (**d**) J-V and (**e**) IPCE characteristics of QDSSCs fabricated with the as-prepared CEs of CNT/metal-sulfides under 1 sun illumination (AM 1.5 G, 100 mW cm^−2^). (**f**) Comparison of power conversion efficiency variation with aging time of QDSSCs based on different CEs. The points are average values of five cells, and the error bars represent standard deviation.

Furthermore, the specific surface area and porosity of the CNT/metal-sulfides were measured by nitrogen adsorption/desorption analysis. CNT/NiS film exhibits a higher Brunauer–Emmett–Teller (BET) surface area (222.5 m^2^ g^−1^) than the CNT/CoS (147.2 m^2^ g^−1^), CNT/CuS (106.3 m^2^ g^−1^), CNT/PbS (29.6 m^2^ g^−1^) and CNT (12.5 m^2^ g^−1^) films, respectively (Fig. 5(a)). Moreover, CNT/NiS has the pore size distribution of 12.5 and 16.1 nm, whereas CNT, CNT/PbS, CNT/CuS and CNT/CoS show the pore sizes of 13.5, 15.6, 12.5–16.8 and 12.5–1.9 nm (Fig. S2†, Supporting Information), indicates the existence of mesoporous structures. These results declare that the CNT/metal-sulfide films having a large surface area with mesoporous structures that is much required for the electrochemical applications.

To examine the mechanism for the charge transfer behavior from CE to the electrolyte, EIS was conducted in CNT/metal-sulfide based symmetrical cells. The Nyquist plots in Fig. 5(b) show two semicircles illustrating the impedance behavior of the symmetric cell. The series resistance (R_s_) was extracted at the high frequency intercept on the real axis of the plot, while the first semicircle in the middle region originated from the charge transfer resistance (R_ct_) and the corresponding chemical capacitance (C_μ_) at the interface of CE/electrolyte. The linear portion of the small circle in the low-frequency region is related to the Warburg diffusion impedance (Z_W_) with in the electrolyte^45^,^46^. The Nyquist plots were fitted by the equivalent circuit demonstrated in the inset of Fig. 5(b), and the corresponding parameters are listed in Table 1.

The R_s_ value of CNT/NiS CE (6.11 Ω) was much smaller than the CNT (10.06 Ω), CNT/PbS (8.51 Ω), CNT/CuS (7.72 Ω), and CNT/CoS (7.17 Ω), respectively. The smallest R_s_ value of CNT/NiS was assigned to the improved conductivity of Ni-foam with CNT/NiS than other CEs. The reduced R_s_ value could lead to an improved FF of the QDDSCs^47^. The difference in electrocatalytic activity among the CEs is associated mainly with the R_ct_ and Z_w_ values. The sample of CNT/NiS exhibited smaller R_ct_ (13.85 Ω) and Z_w_ (5.02 Ω) values than the samples of CNT (R_ct_ = 59.05 Ω, Z_w_ = 10.05 Ω), CNT/PbS (R_ct_ = 47.52 Ω, Z_w_ = 9.78 Ω), CNT/CuS (R_ct_ = 31.49 Ω, Z_w_ = 7.84 Ω), and CNT/CoS (R_ct_ = 26.02 Ω, Z_w_ = 5.02 Ω), showing that the CNT/NiS electrode exhibits enhanced electrocatalytic activity toward polysulfide reduction. This activity might have been due to the higher surface area because of the uniform agglomerated NiS nanoparticle distribution on the CNT network, as well as to the large reduction activity of NiS. This indicates that the CNT/NiS electrode can effectively catalyze the reduction of the polysulfide electrolyte due to the low R_ct_ at the interface of the CE/electrolyte, leading to the high performance of the QDSSC with CNT/NiS CEs.

To reconfirm the electrocatalytic activity of the CNT/metal-sulfide composite catalysts, Tafel polarization curves were measured using symmetrical cells. Figure 5(c) shows the Tafel curves with the logarithmic current density as a function of the voltage in the polysulfide electrolyte. The exchange current density (J_0_) can be extracted from the extrapolated intercepts of the anodic and cathodic branches of the related Tafel curves, thus, the slope of the cathodic and anodic branches indicates J_0_. The slope of the composite CEs varied in the following order: CNT/NiS > CNT/CoS > CNT/CuS > CNT/PbS > CNT, indicating a higher and lower J_0_ for the CNT/NiS and CNT electrode surface. The variation of J_0_ values is consistent with the R_ct_ values obtained in the EIS measurements and both values are related to the following equation (1):


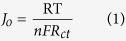


where T is the absolute temperature, R is the gas constant, F is the Faraday constant, n is the number of electrons participating in the electrochemical reduction reaction, and R_ct_ is the charge transfer resistance extracted from the EIS results. This result confirms the higher catalytic activity of CNT/NiS CE than the other CEs. Another important factor inferred from the Tafel curve at a high potential is the limiting diffusion current density (J_lim_). The J_lim_ value is connected directly to the diffusion coefficient (D) of the polysulfide electrolyte using equation (2):


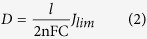


where D is the diffusion coefficient of the polysulfide, C is the concentration of S_n_^2−^, l is the spacer thickness, and n and F have their usual meanings. The J_lim_ for the CNT/NiS CE is higher than that for CNT and other CNT/metal-sulfide CEs, which demonstrates that CNT/NiS has a larger diffusion velocity of S^2−^/S_n_^2−^ in the polysulfide redox couple, which is a magnificent advantage for the electrocatalytic activity of CE. The high J_0_ and J_lim_ values of CNT/NiS and CNT/CoS CEs would favor efficient electron transfer at the CE/electrolyte interface, and result in higher J_SC_, FF, and photovoltaic performance in the QDSSCs.

QDSSCs were characterized under the standard simulated AM 1.5 illumination with an intensity of 100 mW cm^−2^. Figure S3† presents the photovoltaic performance of the QDSSCs assembled with bare metal sulfide CEs, and Table S2 lists the corresponding performance parameters. The QDSSC with the NiS CE shows a higher power conversion efficiency (η) of 3.06% than PbS (2.13%), CuS (2.44%), and CoS (2.80%). To further enhance the performance of QDSSCs, metal sulfides were attached to the surface of the CNTs. As a result, Fig. 5(d) shows the J-V characteristics of the QDSSCs extracted by the various composite CNT/metal-sulfide CEs fabricated in the present study, and Table 1 lists the photovoltaic parameters. The QDSSC designed with CNT CE exhibited a short-current density (J_sc_) of 10.19 mA cm^−2^, an open-circuit voltage (V_oc_) of 0.582 V, and a fill factor (FF) of 0.567, yielding an overall power conversion efficiency (η) of 3.36%. The low performance originated from the low electrocatalytic activity of the CNT CEs toward the reduction of polysulfide, despite the large electrical conductivity of CNT. Therefore, metal sulfides (PbS, CuS, CoS, and NiS) were deposited on the CNT network to support the increased catalytic activity of the CE, which offers an elevated performance of QDSSCs. As a result, the QDSSC with CNT/PbS CE produced a J_sc_, FF, and η of 12.50, 0.578, and 4.27%, respectively. In contrast, the CNT/CuS composite CE-based QDSSC had a J_sc_, FF, and η of 14.50, 0.581, and 5.05%, respectively, and the CNT/CoS composite CE-based QDSSC had a moderate J_sc_, FF, and η of 16.14, 0.588 and 5.78%, respectively. Interestingly, when NiS was deposited on the CNT (CNT/NiS), the J_sc_ and FF were increased greatly with 17.53 mA cm^−2^ and 0.595, respectively, and the highest η of 6.41% was obtained, which is much higher than that of the CNT (3.36%), CNT/PbS (4.27%), CNT/CuS (5.05%), and CNT/CoS (5.78%) composite CEs. In accordance with Tafel and EIS analyses, the tendency of the electrocatalytic activities of the CEs was in the order of CNT/NiS > CNT/CoS > CNT/CuS > CNT/PbS > CNT. This tendency agrees with that of the η values of their QDSSCs. This remarkable increase in the FF and η of QDSSCs with CNT/NiS CE is apparently due to the higher electrical conductivity, the best electrocatalytic ability toward S_n_^2−^ reduction and the improved charge transfer ability. To show the reproducibility, more than five cells assembled with CNT/metal-sulfide CEs were fabricated and the results are shown in Table S3. To the best of our knowledge, it is worth denoting that the results of CNT/CoS and CNT/NiS are superior to that of the recent reports of carbon based composite CEs (Table 2). The photocurrent response to the incident light for QDSSCs with various CEs was characterized by IPCE analysis, as shown in Fig. 5(e). The IPCE values of the QDSSCs with the composite CEs containing CNT, CNT/PbS, CNT/CuS, CNT/CoS, and CNT/NiS over a frequency range of 400–650 nm were approximately 58%, 64%, 73%, 78%, and 84%, respectively. The significant IPCE improvement of the CNT/NiS CE-based QDSSCs also suggests that CNT/NiS possesses super-electrocatalytic activity in reducing S_n_^2−^ to nS^2−^.

The stability of a QDSSC is important factor in real applications. The stability of sealed QDSSCs with composite CEs was investigated under continuous illumination with AM 1.5 G simulated sun light for over a 50 h period. The power conversion efficiencies were observed over a five hour interval, as shown in Fig. 5(f). The η of all CEs showed a steady increase in the first few hours, which was attributed to the capillary effect of a slow permeation of the electrolyte solution into the pores of TiO_2_, and enhanced ionic transport due to heating of the electrolyte^48^. After continuous observations for 50 h, the η of QDSSCs based on the CNT, CNT/PbS, CNT/CuS and CNT/CoS CEs retained 78.57%, 94.14%, 88.11%, and 96.71% of its initial value, whereas the CNT/NiS showed a 4.83% increase compared to its initial value (6.41% to 6.72%). This shows that the CNT/NiS-based QDSSC has high chemical stability and superior corrosion resistance compared to that of CNT, CNT/PbS, CNT/CuS, and CNT/CoS based QDSSCs.

**Figure 6:**
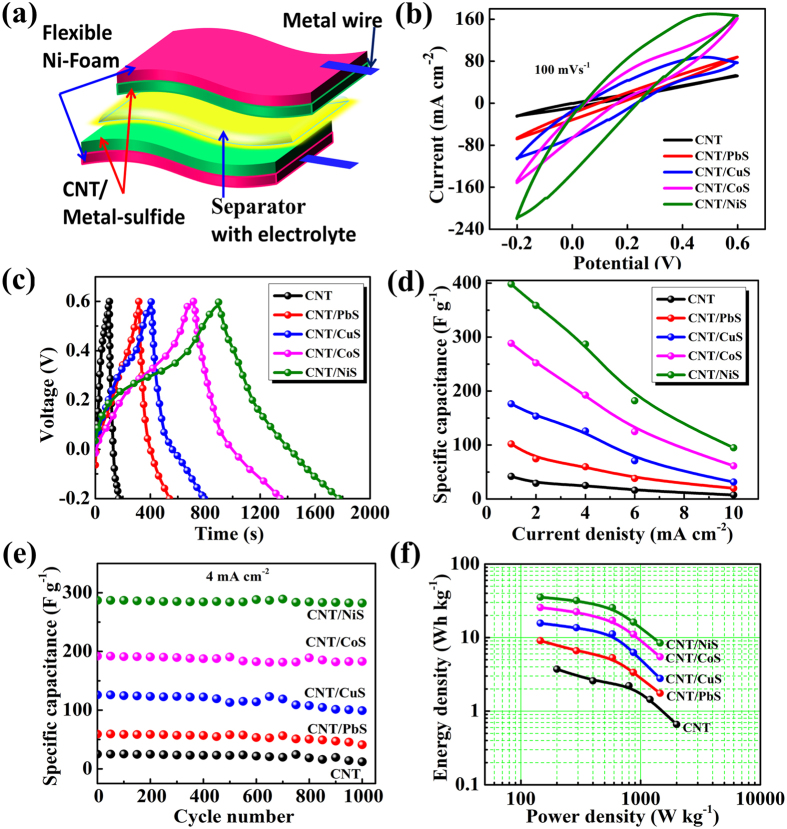
(**a**) Schematic of the flexible symmetric supercapacitor. (**b**) CV graphs for symmetric CNT/metal-sulfide supercapacitor devices at 100 mV s^−1^ in polysulfide electrolyte. (**c**) Charge-discharge curve at 1 mA cm^−2^, (**d**) specific capacitance versus current density, (**e**) stability performance and (**f**) Ragone plot for symmetric CNT/metal-sulfide supercapacitor cells.

In addition to applications for the CE in QDSSCs, CNT/metal-sulfides can also be applicable for energy storage devices. To assess the CNT and CNT/metal-sulfides as supercapacitor electrodes, a symmetrical two-electrode cell configuration was used in a polysulfide electrolyte. The CNT/metal-sulfides were deposited on a piece of Ni-foam (1 × 3 cm^2^). For the cyclic voltammetry (CV) and charge-discharge measurements, two electrode symmetrical cells were prepared with a positive and negative electrode containing an equal amount of active material. The mass loading of active material on the CNT, CNT/PbS, CNT/CuS, CNT/CoS, and CNT/NiS electrodes was 5.0, 5.5, 5.5, 5.5, and 5.5 mg, respectively. The two electrodes were separated by cellulose paper soaked with electrolyte (polysulfide) and pressed, then wrapped with parafilm. The schematic of the flexible symmetric supercapacitor is shown in Fig. 6(a). The specific capacitance (C_s_), energy density (E, W h kg^−1^), and power density (P, W kg^−1^) were obtained from charge-discharge analysis using the following equations:


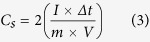



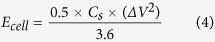



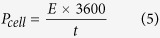


where I (A), t (s), 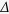
V (V), and m (g) are the discharge current, discharge time, potential window, and mass of the active material, respectively. CV was performed over the potential range, -0.2 to + 0.6 V, at different scanning rates. The galvanostatic charge-discharge tests were conducted at various constant currents at voltages between −0.2 and +0.6 V.

Figure 6(b) shows the typical CV scans of the CNT and CNT/metal-sulfide samples at a scan rate of 100 mV s^−1^ over the potential range of −0.2 to 0.6 V. The CV areas of the electrodes increased with increasing scan rate, indicating good capacitance retention (Fig. S4†, Supporting Information). The CV curves in the current work showed no obvious redox peaks, denoting that the present supercapacitors are charged and discharged at a pseudoconstant rate^49^. Therefore, the two-electrode symmetric supercapacitors are considered to be mainly nonfaradaic within their respecting voltage range^50^, which has been observed in many symmetric supercapacitors^50^,^51^. The CNT supercapacitor showed tilted CVs with a low area, illustrating inferior capacitive behavior. The supercapacitors, fabricated using CNT/PbS, CNT/CuS, and CNT/CoS showed reasonable CV areas, indicating sufficient capacitance. The electrodes prepared using CNT/NiS showed a larger CV area, suggesting a higher capacitance. The high performance of the CNT/NiS electrode was attributed to the material with a high specific surface area and high porosity, which improves the transport of ions to the active sites of the electrodes^52^.

Galvanostatic charge–discharge (GCD) is the most accurate technique for capacitance measurements, hence, GCD measurements were conducted for the CNT and CNT/metal-sulfides samples. Figure S5† shows the typical GCD measurements of CNT and CNT/metal-sulfide samples in a two-electrode configuration at various current densities ranging from 1 to 10 mA cm^−2^ with voltages between −0.2 and 0.6 V. Figure 6(c) presents the GCD curves for the CNT, CNT/PbS, CNT/CuS, CNT/CoS, and CNT/NiS at a current density of 1 mA cm^−2^. The CNT/NiS exhibited the longest discharge duration compared to the CNT, CNT/PbS, CNT/CuS, and CNT/CoS, reflecting the substantially superior performance of CNT/NiS. The specific capacitance of the CNT/NiS device was 398.16 F g^−1^ at 1 mA cm^−2^ from GCD curve, whereas specific capacitances of 288.43, 176.20, 101.94, and 41.87 F g^−1^ were observed in the CNT/CoS, CNT/CuS, CNT/PbS, and CNT devices, respectively (Fig. 6(d)). These results show that the specific surface area and mesoporous structures with a uniform morphology are crucial factors for obtaining high supercapacitor performance. In addition, the stability of the symmetric CNT/metal-sulfide cell was examined by repeated charge-discharge testing at a current density of 4 mA cm^−2^ for 1000 cycles (Fig. 6(e)). The cycling stability of the CNT/NiS symmetric cell shows that the capacitance retention is approximately 98.39% of its initial value after 1000 cycles, which is better than the CNT/CoS (95.3%), CNT/CuS (78.57%), CNT/PbS (69.49%), and CNT (48%) cells. Furthermore, Fig. 6(f) shows a Ragone plot of the specific energy vs. the specific power, which was used to estimate the precise performance of the supercapacitors. The maximal energy density of 35.39 W h kg^−1^ and power density of 145.45 W kg^−1^ of the CNT/NiS cell was superior to that of CNT/CoS (25.63 W h kg^−1^ and 145.45 W kg^−1^), CNT/CuS (15.66 W h kg^-1^ and 145.45 W kg^−1^), CNT/PbS (9.06 W h kg^−1^ and 145.45 W kg^−1^), and CNT (3.72 W h kg^−1^ and 199.80 W kg^−1^). This confirms that CNT/NiS is the best supercapacitor cell among the other supercapacitor cells fabricated from CNT/metal-sulfides. The CV and GCD curves of CNT/NiS symmetric supercapacitor extracted at various bending angles show nearly the same capacitive behavior (Fig. S6†), demonstrating no apparent change of electrochemical behavior at different bending angles. These results denote that the CNT/NiS device is highly flexible.

## Conclusion

In conclusion, novel composite CNT/metal-sulfides as electrodes were designed for energy conversion (QDSSCs) and energy storage (SCs) applications. CNT/metal-sulfide composites were first synthesized by grinding a mixture of organic binders and CNTs. The resulting slurry was then pasted onto Ni-foam. Metal-sulfides were then deposited on the surface of the CNTs by a facile solution approach. The roles of the metal sulfides were to provide active sites for the reduction of the polysulfide redox couple, while the CNT network facilitates the electron pathways to metal sulfides. As a result, a new catalyst of the CNT/NiS composite CE achieved a power-conversion efficiency of up to 6.41% for the QDSSCs, indicating a 90.8%, 50.1%, 27.0%, and 10.9% increase in the η value compared to the devices prepared using the CNT, CNT/PbS, CNT/CuS, and CNT/CoS CEs, respectively. The QDSSC-based on CNT/NiS CE delivered excellent stability. The EIS and Tafel polarization measurements supported the excellent electrocatalytic activity of the CNT/NiS composite CE. In addition, CNT/metal-sulfide composites were applied to develop a symmetric supercapacitor using a polysulfide electrolyte. The CNT/NiS symmetrical supercapacitor exhibited a high specific capacitance and energy density of 398.16 F g^−1^ and 35.39 W h kg^−1^ at 1 mA cm^−2^, and enhanced cycling stability (98.39% retention after 1000 cycles at 4 mA cm^−2^). Overall, these results indicate that the CNT/metal-sulfide composites provide a new path for the development of similar advanced electrochemical electrode materials for a range of applications. Fourth, the flexible property of the supercapacitor is highly desired for actual applications.

## Methods

### Materials

Nickel sulfate hexahydrate (NiSO_4_.6H_2_O), lead(II) nitrate (Pb(NO_3_)_2_), cobalt chloride hexahydrate (CoCl_2_.6H_2_O), copper sulfate pentahydrate (CuSO_4_·5H_2_O), selenium (Se), zinc acetate dehydrate [Zn(CH_3_COO)_2_.2H_2_O], thioacetamide (C_2_H_5_NS), urea (CH_4_N_2_O), acetic acid (C_2_H_4_O_2_), cadmium acetate dehydrate [Cd(CH_3_COO)_2_.2H_2_O], sulfur (S), potassium chloride (KCl), sodium sulfide (Na_2_S), cadmium sulfate (CdSO_4_·8/3H_2_O), nitro tri-acetic acid (N(CH_2_CO_2_H)_3_), sodium thiosulfate (Na_2_S_2_O_3_), N-methyl-2-pyrrolidone (C_5_H_9_NO), polyvinylidene fluoride (PVDF), and Ni-foams were purchased from Sigma-Aldrich and used without further purification. TiO_2_ paste (Ti-Nanoxide HT/SP) was supplied by Solaronix. The MWCNTs (Carbon Nano-Material Technology Co., Ltd) were used as received.

### Synthesis of CNT/metal sulfide composites

Prior to synthesis, the Ni foam was etched ultrasonically with a 2 M HCl solution for 30 min and then cleaned sequentially with acetone, ethanol, and deionized water (DI) for 10 min each. The counter electrode was prepared by mixing 0.05 g of MWCNT powder and 0.1 g of polyvinylidene fluoride (PVDF) in a solvent of 2 ml N-Methyl-2-pyrrolidone (NMP). The mixture was first well mixed with a mortar and pestle to form a slurry and coated onto a piece of Ni foam (1.3 × 1.6 cm^2^). The coated foam was then sintered at 150 °C for 30 min, the fabricated thin film is denoted as the CNT electrode.

A facile solution approach of chemical bath deposition (CBD) was used to deposit metal sulfides on the Ni-foam based CNT electrode. The CuS, NiS, PbS, CoS deposition solutions were prepared with 0.1 M CuSO_4_·5H_2_O (or NiSO_4_.6H_2_O, Pb(NO_3_)_2_, and CoCl_2_.6H_2_O) as the metal source and 0.4 M C_2_H_5_NS as the S source in 50 ml deionized water. Subsequently, 0.6 M C_2_H_4_O_2_ was added drop-wise to the CuS and NiS solutions, and 0.4 M CH_4_N_2_O was added to the CoS and PbS solutions. Composite electrodes were prepared by dipping CNT electrodes vertically into the CuS, NiS, PbS and CoS solutions, and deposition was carried out for 90 min at 90 °C. After deposition, the electrodes were removed from the oven and cleaned with DI water and ethanol. Finally, the prepared materials were dried overnight at 100 °C prior to use. The samples denoted as CNT/PbS, CNT/CuS, CNT/CoS, and CNT/NiS were prepared from PbS, CuS, CoS, and NiS solutions, respectively.

### TiO_2_/CdS/CdSe/ZnS photoanode fabrication

Mesoporous TiO_2_ films were prepared by doctor-blading a TiO_2_ paste onto the fluorine-doped tin oxide (FTO) substrate with an active area of 0.27 cm^2^, followed by sintering at 450 °C for 30 min to remove impurities and improve the crystallinity. CdS seed layer was deposited by SILAR process on surface of TiO_2_ to facilitate the subsequent CdSe growth. The TiO_2_ film was first immersed into a 0.1 M Cd(CH_3_COO)_2_.2H_2_O aqueous solution for 2 min and rinsed with DI water and ethanol, after the film was immersed into a 0.1 M Na_2_S aqueous solution for another 2 min, followed by rinsing with DI water and ethanol and dry with drier. This process was repeated five times. The as prepared samples names as TiO_2_/CdS.

CdSe QDs was deposited on the surface of TiO_2_/CdS film through a CBD procedure. TiO_2_/CdS films dipped into a solution containing mixture of 80 mM CdSO_4_·8/3H_2_O, 90 mM N(CH_2_CO_2_H)_3_, and 80 mM Na_2_SeSO_3_ at 40 °C for 135 min. The electrodes were annealed at 300 °C for 1 h, cooled naturally. The as-prepared electrodes are named as TiO_2_/CdS/CdSe. After CdSe deposition, 3 cycles of ZnS was deposited by a SILAR method through dipping the TiO_2_/CdS/CdSe film in an aqueous solution containing 0.1 M Zn(CH_3_COO)_2_.2H_2_O and 0.1 M Na_2_S for 1 min. Finally the photoanode is denoted as TiO_2_/CdS/CdSe/ZnS.

### QDSSC device fabrication

The preparation process for the TiO_2_/CdS/CdSe/ZnS photoanode is shown in the supporting information. The QDSSCs were assembled as a sandwich structure with a photoanode and a CE by a sealant (SX 1170–60, Solaronix) at 100 °C. The polysulfide electrolyte (1 M Na_2_S, 2 M S, and 0.1 M KCl in methanol: water is 7:3) was used to fill the space between the electrodes. The back of the porous Ni foam-based CNT/metal-sulfides were made up with a piece of glass sheet, so electrolyte leakage did not occur through the porous CNT/metal-sulfide CE.

### Fabrication of symmetric cells for Tafel polarization and EIS measurements

Tafel polarization measurements and EIS experiments were conducted in the dark using a symmetrical dummy cell (CE/electrolyte/CE) configuration of a symmetrical cell with an active area of 0.27 cm^2^. The EIS measurements were performed over the frequency range, 500 kHz-0.1 Hz, at zero bias with a 10 mV AC amplitude. The Tafel polarization measurements were conducted at a scan rate of 10 mV s^−1^.

### Characteriations

The surface morphology and structure of the resulting samples were studied by field emission scanning electron microscopy (FE-SEM, S-2400, Hitachi) with energy-dispersive X-ray spectroscopy (EDX, 15 kV). The crystal structure was examined by X-ray diffraction (XRD) analysis (D8 ADVANCE with a DAVINCI diffractometer (Bruker AXS)) with Cu Kα radiation operated at 40 kV and 40 mA. The chemical composition was analyzed by X-ray photoelectron spectroscopy (XPS, VG Scientific ESCALAB 250). Transmission electron microscopy (TEM) and high-resolution TEM (HRTEM) were carried out on a CJ111 high-resolution electron microscope with an acceleration voltage of 200 kV. Brunauer–Emmett–Teller (BET) analysis was used to measure the specific surface area of the samples using a BELsorp-Max (BEL, Japan) instrument at 77 K.

The current-voltage (J-V) curves of the QDSSCs were derived using an ABET Technologies (USA) solar simulator under one sun illumination (AM 1.5 G, 100 mW cm^−2^). The incident photon-to-current conversion efficiency (IPCE) spectra of the QDSSCs were examined using an Oriel^®^ IQE-200™. Electrochemical impedance spectroscopy (EIS) and Tafel polarization were executed on symmetrical cells using a BioLogic potentiostat/galvanostat/EIS analyzer (SP-150, France). CV was carried out in a symmetrical supercapacitor using a BioLogic electrochemical analyzer. Supercapacitor charging-discharging measurements were carried out using galvanostatic charge-discharge (GCD, BioLogic analyzer, SP-150, France) tests.

## Additional Information

**How to cite this article:** Muralee Gopi, C. V. V. *et al*. Carbon nanotube/metal-sulfide composite flexible electrodes for high-performance quantum dot-sensitized solar cells and supercapacitors. *Sci. Rep.* **7**, 46519; doi: 10.1038/srep46519 (2017).

**Publisher's note:** Springer Nature remains neutral with regard to jurisdictional claims in published maps and institutional affiliations.

## Supplementary Material

Supporting Information

## Figures and Tables

**Table 1:** Summary of the photovoltaic parameters of TiO_2_/CdS/CdSe/ZnS QDSSC based CNT/metal-sulfide CEs and extracted parameters from EIS based on symmetrical dummy cells.

CEs	V_OC_ (V)	J_SC_ (mA cm^−2^)	FF	η %	R_s_ (Ω)	R_ct_ (Ω)	Z_w_ (Ω)
CNT	0.582 ± 0.002	10.19 ± 0.06	0.567 ± 0.004	3.36 ± 0.05	10.06	59.05	10.15
CNT/PbS	0.590 ± 0.005	12.50 ± 0.08	0.578 ± 0.003	4.27 ± 0.04	8.51	47.52	9.78
CNT/CuS	0.599 ± 0.004	14.50 ± 0.1	0.581 ± 0.004	5.05 ± 0.02	7.72	31.49	7.84
CNT/CoS	0.609 ± 0.005	16.14 ± 0.07	0.589 ± 0.003	5.78 ± 0.03	7.17	26.02	5.52
CNT/NiS	0.614 ± 0.006	17.53 ± 0.09	0.595 ± 0.002	6.41 ± 0.02	6.11	13.85	5.02

The average and deviations are calculated from 5 devices.

**Table 2:** QDSSC performance comparison with recent reports of composite counter electrodes.

Counter electrode	V_OC_ (V)	J_SC_ (mA cm^−2^)	FF	η %	References
Mo_2_N/CNT-RGO	0.68	16.93	0.47	5.41	Adv. Energy Mater. **2014**, 4, 1300775
CuS/EC	0.521	14.60	0.507	3.86	J. Phys. Chem. C **2014**, 118, 16526
MWCNT-CZTSe	0.53	17.04	0.51	4.60	Nanoscale, **2013**, 5, 6992
Cu_1.18_S–GOR	0.520	18.04	0.58	5.42	Nanoscale, **2016**, 8, 10632
CuInS_2_:C	0.512	14.16	0.60	4.32	ACS Appl. Mater. Interfaces **2013**, 5, 5954
Cdot-Au NR	0.708	16.6	0.46	5.4	Carbon **2016**, 96, 139
RGO-Cu_2_S	0.526	15.08	0.44	3.49	Adv. Energy Mater. **2014,** 4, 1301564
CNT/CoS	**0.609**	**16.14**	**0.589**	**5.78**	**This Work**
CNT/NiS	**0.614**	**17.53**	**0.595**	**6.41**	**This Work**
