# Supplementary material for: Carbon nanotube/metal-sulfide composite flexible electrodes for high-performance quantum dot-sensitized solar cells and supercapacitors
Source: Sci Rep. 2017 Apr 19;7:46519. doi: 10.1038/srep46519 (PMC5395955; doi:10.1038/srep46519)
Supplement: Supporting Information [file srep46519-s1.doc]

Supporting Information

Carbon nanotube/metal-sulfide composite flexible electrodes for high-performance quantum dot-sensitized solar cells and supercapacitors

Chandu V.V. Muralee Gopi,^a^ Seenu Ravi,^b^ S. Srinivasa Rao,^a^

Araveeti Eswar Reddy ^a^ and Hee-Je Kim^a^*

*^a^ School of Electrical Engineering, Pusan National University, Gumjeong-Ku, Jangjeong-Dong, Busan 46241, South Korea*

*^b^ Department of Chemical Engineering, Inha University, Incheon, 22212, South Korea.*

**Corresponding authors:**

E-mail: [heeje@pusan.ac.kr](mailto:heeje@pusan.ac.kr) (H.-J. Kim)

**Table S1.** Element composition of CNT/metal-sulfides from XPS analysis.

| **Thin films** | **CNT** | **CNT/PbS** | **CNT/CuS** | **CNT/CoS** | **CNT/NiS** |
| --- | --- | --- | --- | --- | --- |
| **C (at%)** | 44.40 | 31.69 | 28.85 | 24.98 | 18.01 |
| **O (at%)** | 48.03 | 13.84 | 8.97 | 6.32 | 6.18 |
| **Pb (at%)** | - | 9.91 | - | - | - |
| **Cu (at%)** | - | - | 9.67 | - | - |
| **Co (at%)** | - | - | - | 9.25 | - |
| **Ni (at%)** | - | - | - | - | 9.14 |
| **S (at%)** | - | 6.91 | 7.99 | 20.6 | 23.47 |


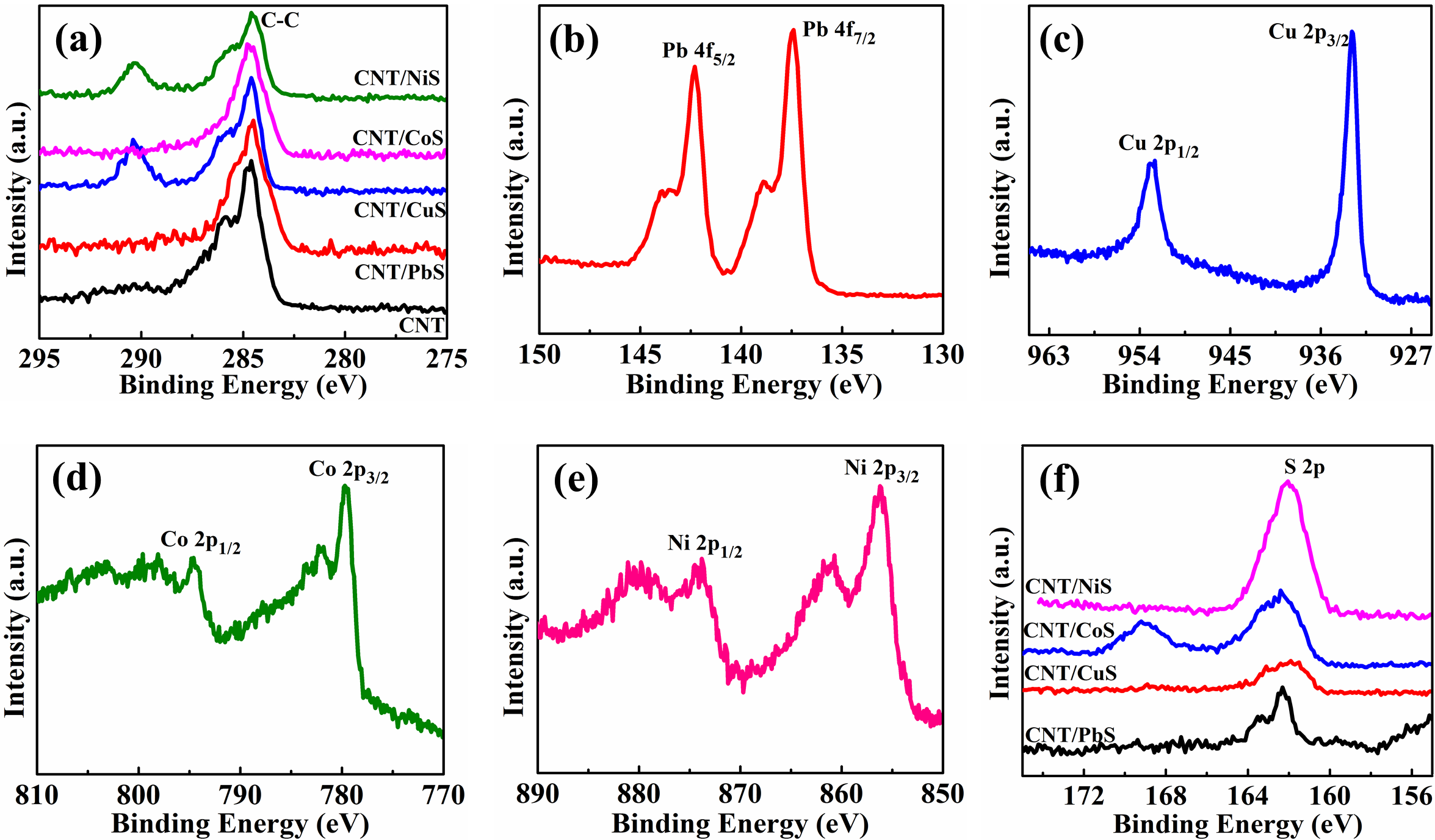


**Figure S1** High resolution scan of the (a) C peak in all electrodes, (b) Pb 4f peak in CNT/PbS, (c) Cu 2p peak in CNT/CuS, (d) Co 2p peak in CNT/CoS, (e) Ni 2p peak in CNT/NiS and (c) S2p peak in all electrodes.


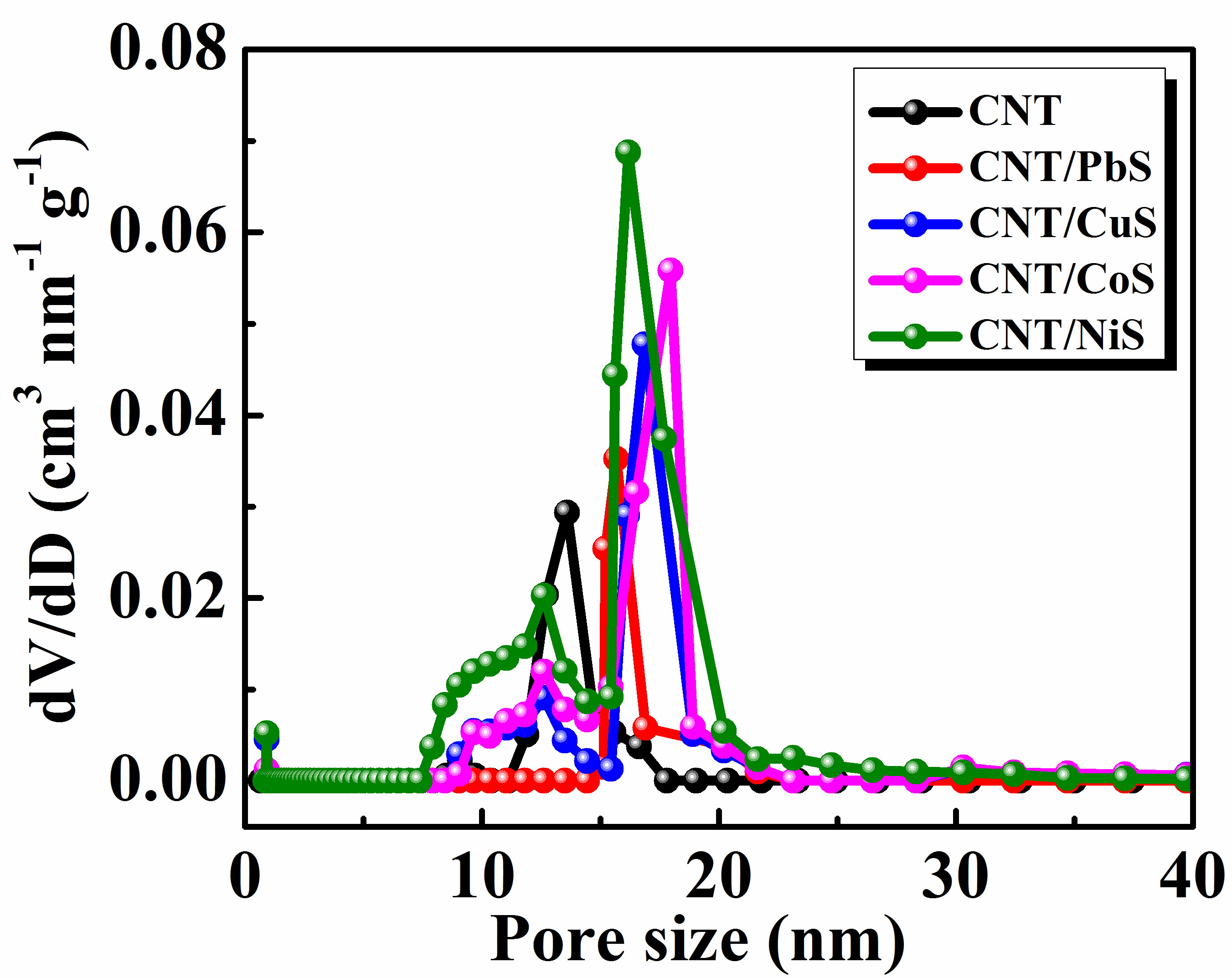


**Figure S2** Pore size distribution of CNT/metal-sulfide electrodes from BET analysis.


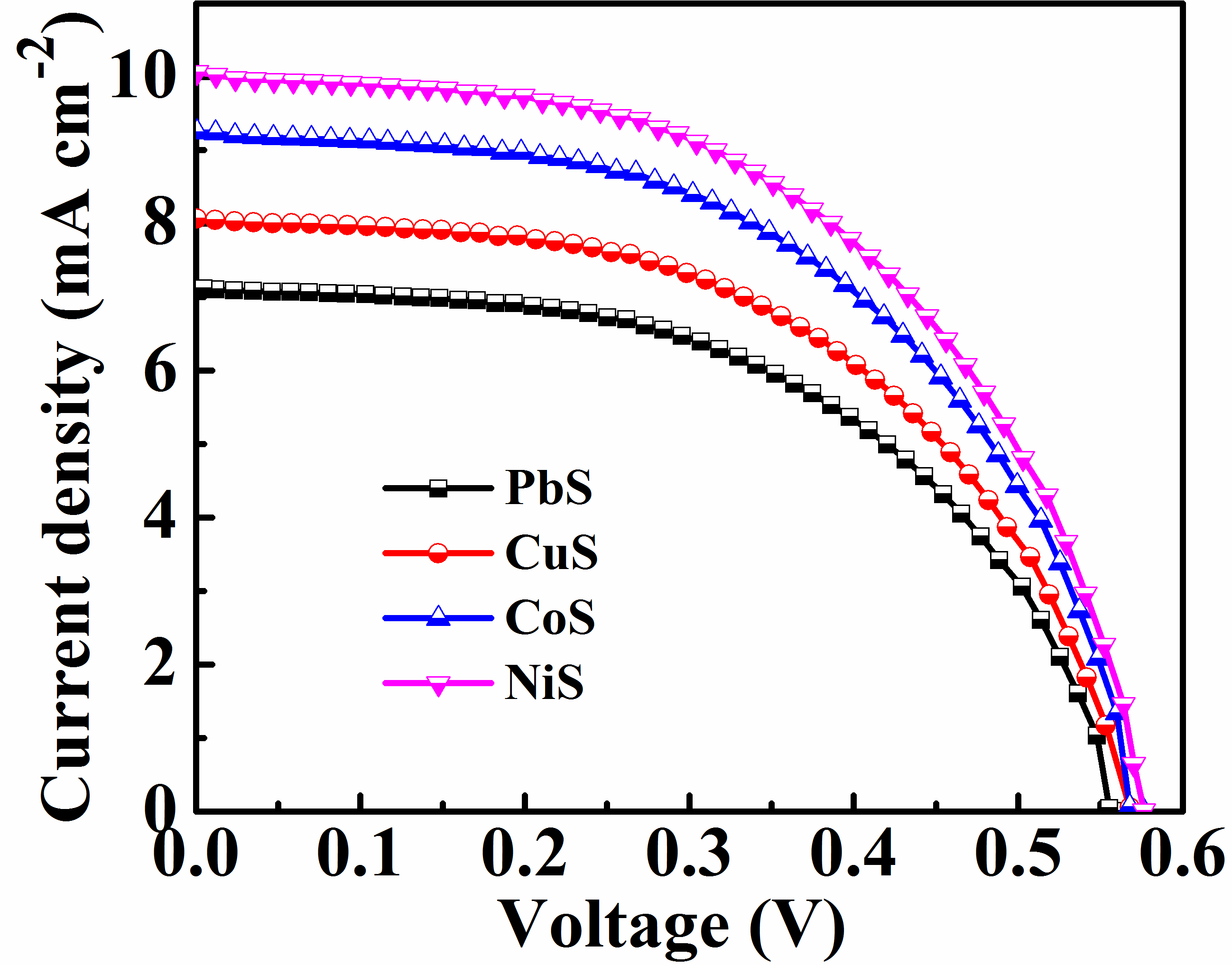


**Figure S3** J–V characteristics for the QDSSCs assembled with the bare metal sulfide CEs under simulated solar illumination at 100 mW cm^-2^.

**Table S2.** Photovoltaic parameters of the QDSSCs based on the bare metal sulfide counter electrodes (PbS, CuS, CoS and NiS) in the presence of polysulfide electrolyte.

| **CE** | **V_OC_ (V)** | **J_SC_ (mA cm^-2^)** | **FF** | **η%** |
| --- | --- | --- | --- | --- |
| PbS | 0.563 | 7.21 | 0.525 | 2.13 |
| CuS | 0.569 | 8.13 | 0.528 | 2.44 |
| CoS | 0.576 | 9.31 | 0.522 | 2.80 |
| NiS | 0.577 | 10.11 | 0.526 | 3.06 |

**Table S3.** Solar cell parameters of QDSSC (multiple cells) with various CNT/metal-sulfide CEs

| **Counter Electrode** | **V_OC_ (V)** | **J_SC_ (mA cm^-2^)** | **FF** | **η (%)** |
| --- | --- | --- | --- | --- |
| **CNT (Cell 1)** | 0.580 | 10.16 | 0.567 | 3.35 |
| **CNT (Cell 2)** | 0.581 | 10.21 | 0.568 | 3.37 |
| **CNT (Cell 3)** | 0.582 | 10.19 | 0.567 | 3.36 |
| **CNT (Cell 4)** | 0.584 | 10.25 | 0.571 | 3.42 |
| **CNT (Cell 5)** | 0.576 | 10.14 | 0.567 | 3.33 |
| **CNT/PbS (Cell 1)** | 0.586 | 12.47 | 0.576 | 4.21 |
| **CNT/PbS (Cell 2)** | 0.590 | 12.50 | 0.578 | 4.27 |
| **CNT/PbS (Cell 3)** | 0.596 | 12.58 | 0.580 | 4.31 |
| **CNT/PbS (Cell 4)** | 0.592 | 12.53 | 0.575 | 4.27 |
| **CNT/PbS (Cell 5)** | 0.581 | 12.47 | 0.575 | 4.20 |
| **CNT/CuS (Cell 1)** | 0.598 | 14.51 | 0.580 | 5.04 |
| **CNT/CuS (Cell 2)** | 0.597 | 14.47 | 0.581 | 5.03 |
| **CNT/CuS (Cell 3)** | 0.599 | 14.50 | 0.581 | 5.05 |
| **CNT/CuS (Cell 4)** | 0.606 | 14.54 | 0.575 | 5.07 |
| **CNT/CuS (Cell 5)** | 0.601 | 14.62 | 0.585 | 5.14 |
| **CNT/CoS (Cell 1)** | 0.606 | 16.12 | 0.584 | 5.70 |
| **CNT/CoS (Cell 2)** | 0.604 | 16.08 | 0.589 | 5.72 |
| **CNT/CoS (Cell 3)** | 0.609 | 16.14 | 0.588 | 5.78 |
| **CNT/CoS (Cell 4)** | 0.610 | 16.23 | 0.591 | 5.85 |
| **CNT/CoS (Cell 5)** | 0.607 | 16.15 | 0.586 | 5.75 |
| **CNT/NiS Cell 1)** | 0.612 | 17.54 | 0.597 | 6.42 |
| **CNT/NiS (Cell 2)** | 0.609 | 17.52 | 0.594 | 6.36 |
| **CNT/NiS (Cell 3)** | 0.617 | 17.44 | 0.593 | 6.39 |
| **CNT/NiS (Cell 4)** | 0.614 | 17.53 | 0.595 | 6.41 |
| **CNT/NiS (Cell 5)** | 0.621 | 17.60 | 0.596 | 6.53 |


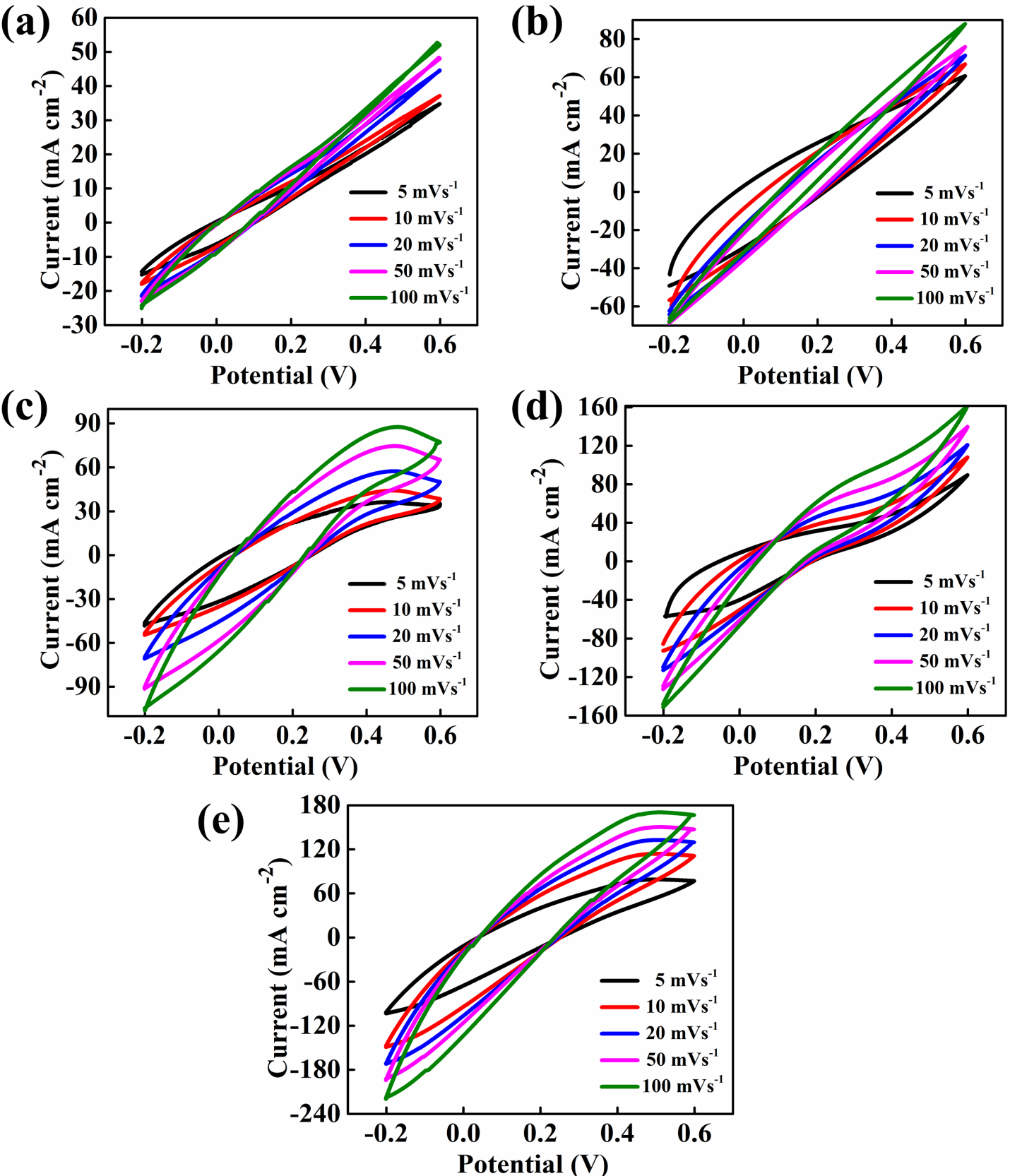


**Figure S4.** CVs of (a) CNT, (b) CNT/PbS, (c) CNT/CuS, (d) CNT/CoS, and (e) CNT/NiS at different scan rates.


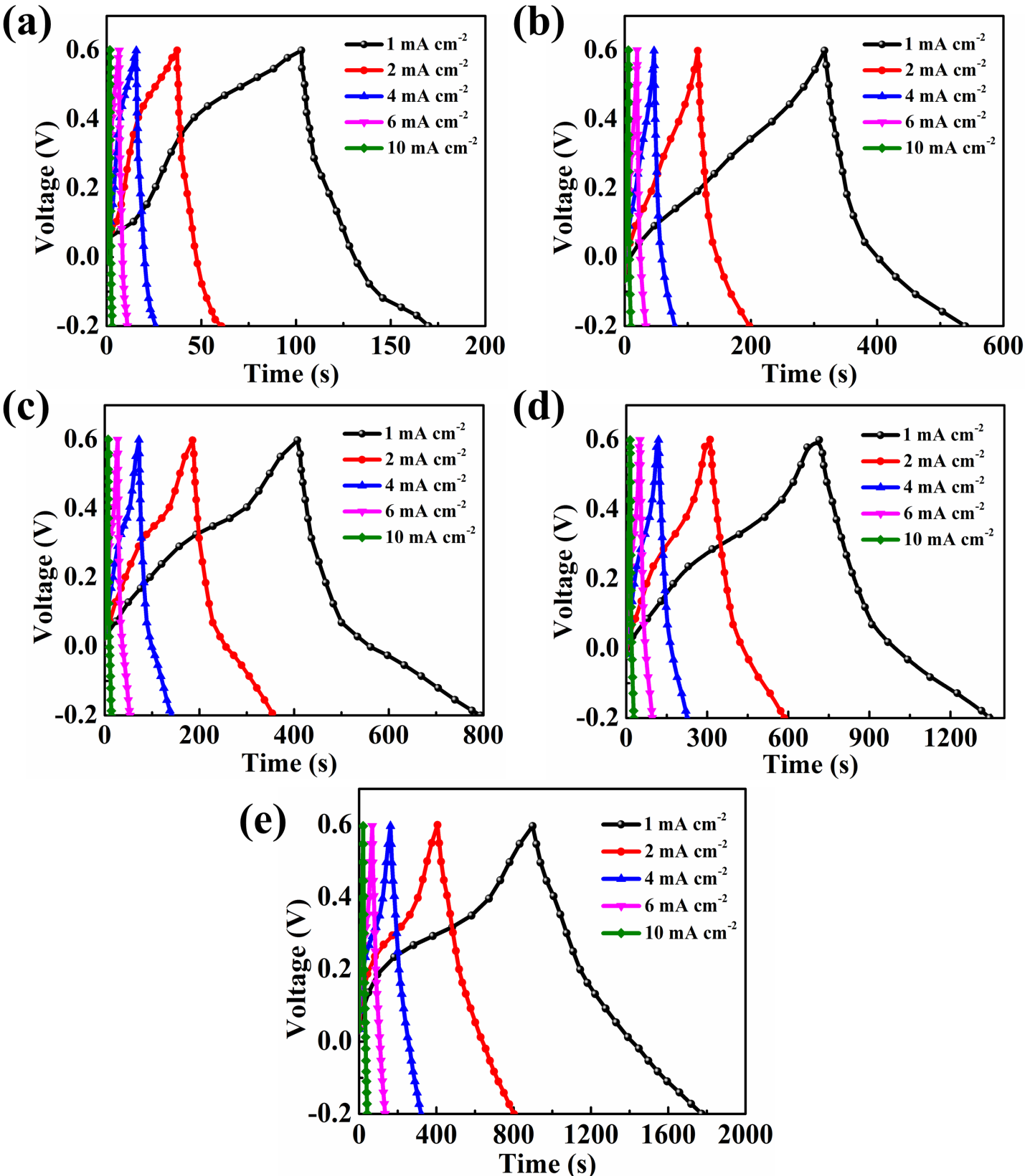


**Figure S5.** Galvanostatic charge-discharge measurements of (a) CNT, (b) CNT/PbS, (c) CNT/CuS, (d) CNT/CoS, and (e) CNT/NiS at different current densities.


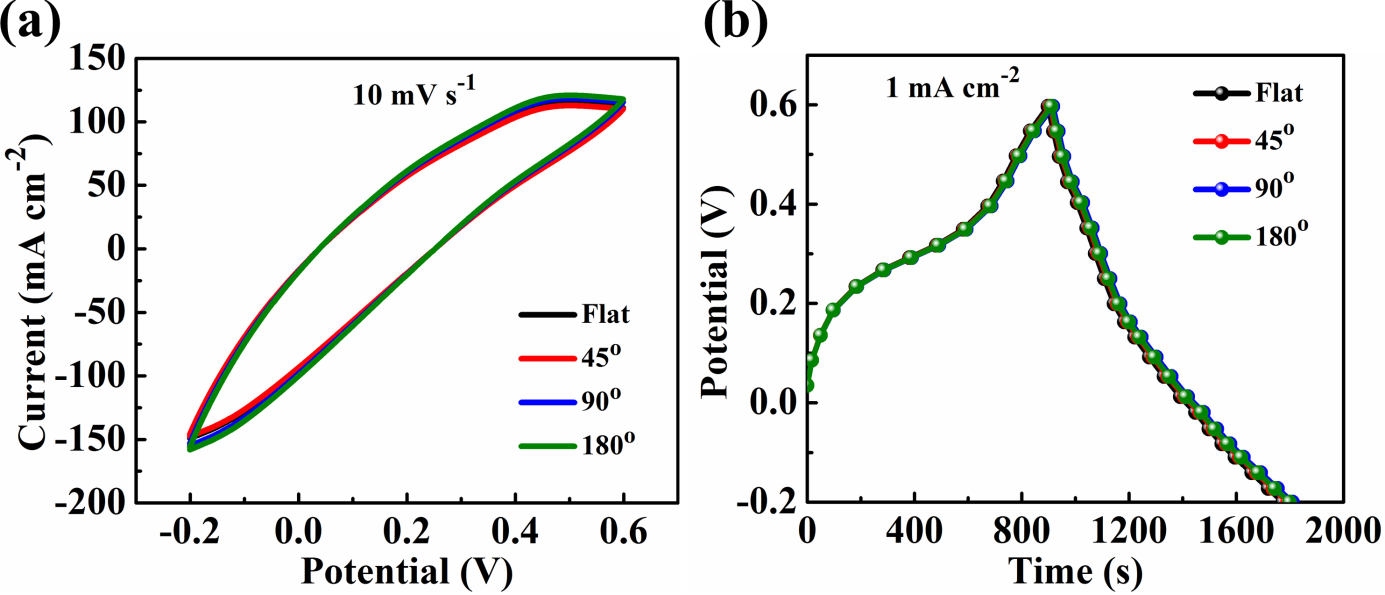


**Figure S6.** Performance of the CNT/NiS symmetric supercapacitor at different bending angles, (a) CV curves at a scan rate of 10 mV s^-1^, and (C) charging–discharging curves taken at 1 mA cm^-2^.
